# Effect of point-of-care C-reactive protein testing on antibiotic prescription in febrile patients attending primary care in Thailand and Myanmar: an open-label, randomised, controlled trial

**DOI:** 10.1016/S2214-109X(18)30444-3

**Published:** 2018-12-13

**Authors:** Thomas Althaus, Rachel C Greer, Myo Maung Maung Swe, Joshua Cohen, Ni Ni Tun, James Heaton, Supalert Nedsuwan, Daranee Intralawan, Nithima Sumpradit, Sabine Dittrich, Zoë Doran, Naomi Waithira, Hlaing Myat Thu, Han Win, Janjira Thaipadungpanit, Prapaporn Srilohasin, Mavuto Mukaka, Pieter W Smit, Ern Nutcha Charoenboon, Marco Johannes Haenssgen, Tri Wangrangsimakul, Stuart Blacksell, Direk Limmathurotsakul, Nicholas Day, Frank Smithuis, Yoel Lubell

**Affiliations:** a Mahidol-Oxford Tropical Medicine Research Unit, Faculty of Tropical Medicine, Mahidol University, Bangkok, Thailand; b Centre for Tropical Medicine and Global Health, Nuffield Department of Clinical Medicine, University of Oxford, Oxford, UK; c Myanmar-Oxford Clinical Research Unit, Yangon, Myanmar; d Medical Action Myanmar, Yangon, Myanmar; e Primary Care Department, Chiangrai Prachanukroh Hospital, Chiangrai, Thailand; f Thai Food and Drug Administration, Ministry of Public Health, Bangkok, Thailand; g Foundation for Innovative New Diagnostics, Geneva, Switzerland; h Department of Medical Research, Yangon, Myanmar

## Abstract

**Background:**

In southeast Asia, antibiotic prescription in febrile patients attending primary care is common, and a probable contributor to the high burden of antimicrobial resistance. The objective of this trial was to explore whether C-reactive protein (CRP) testing at point of care could rationalise antibiotic prescription in primary care, comparing two proposed thresholds to classify CRP concentrations as low or high to guide antibiotic treatment.

**Methods:**

We did a multicentre, open-label, randomised, controlled trial in participants aged at least 1 year with a documented fever or a chief complaint of fever (regardless of previous antibiotic intake and comorbidities other than malignancies) recruited from six public primary care units in Thailand and three primary care clinics and one outpatient department in Myanmar. Individuals were randomly assigned using a computer-based randomisation system at a ratio of 1:1:1 to either the control group or one of two CRP testing groups, which used thresholds of 20 mg/L (group A) or 40 mg/L CRP (group B) to guide antibiotic prescription. Health-care providers were masked to allocation between the two intervention groups but not to the control group. The primary outcome was the prescription of any antibiotic from day 0 to day 5 and the proportion of patients who were prescribed an antibiotic when CRP concentrations were above and below the 20 mg/L or 40 mg/L thresholds. The primary outcome was analysed in the intention-to-treat and per-protocol populations. The trial is registered with ClinicalTrials.gov, number NCT02758821, and is now completed.

**Findings:**

Between June 8, 2016, and Aug 25, 2017, we recruited 2410 patients, of whom 803 patients were randomly assigned to CRP group A, 800 to CRP group B, and 807 to the control group. 598 patients in CRP group A, 593 in CRP group B, and 767 in the control group had follow-up data for both day 5 and day 14 and had been prescribed antibiotics (or not) in accordance with test results (per-protocol population). During the trial, 318 (39%) of 807 patients in the control group were prescribed an antibiotic by day 5, compared with 290 (36%) of 803 patients in CRP group A and 275 (34%) of 800 in CRP group B. The adjusted odds ratio (aOR) of 0·80 (95% CI 0·65–0·98) and risk difference of −5·0 percentage points (95% CI −9·7 to −0·3) between group B and the control group were significant, although lower than anticipated, whereas the reduction in prescribing in group A compared with the control group was not significant (aOR 0·86 [0·70–1·06]; risk difference −3·3 percentage points [–8·0 to 1·4]). Patients with high CRP concentrations in both intervention groups were more likely to be prescribed an antibiotic than in the control group (CRP ≥20 mg/L: group A *vs* control group, p<0·0001; CRP ≥40 mg/L: group B *vs* control group, p<0·0001), and those with low CRP concentrations were more likely to have an antibiotic withheld (CRP <20 mg/L: group A *vs* control group, p<0·0001; CRP <40 mg/L: group B *vs* control group, p<0·0001). 24 serious adverse events were recorded, consisting of 23 hospital admissions and one death, which occurred in CRP group A. Only one serious adverse event was thought to be possibly related to the study (a hospital admission in CRP group A).

**Interpretation:**

In febrile patients attending primary care, testing for CRP at point of care with a threshold of 40 mg/L resulted in a modest but significant reduction in antibiotic prescribing, with patients with high CRP being more likely to be prescribed an antibiotic, and no evidence of a difference in clinical outcomes. This study extends the evidence base from lower-income settings supporting the use of CRP tests to rationalise antibiotic use in primary care patients with an acute febrile illness. A key limitation of this study is the individual rather than cluster randomised study design which might have resulted in contamination between the study groups, reducing the effect size of the intervention.

**Funding:**

Wellcome Trust Institutional Strategic Support Fund grant (105605/Z/14/Z) and Foundation for Innovative New Diagnostics (FIND) funding from the Australian Government.

## Introduction

Southeast Asia is a global hub of antimicrobial resistance, which is associated with high morbidity and mortality,^1^ and is a probable exporter of antimicrobial resistance through dense travel and agricultural trade networks.^2^ With an extensive population reach, primary care could be a major force in the fight against antimicrobial resistance; instead, primary care is a setting in which antibiotic prescription is widespread and poorly regulated.^3^ Challenges include high patient throughput, diagnostic uncertainty, patient expectations of treatment, and reluctance to refrain from prescribing when follow-up is challenging.^4^, ^5^, ^6^ A situational analysis of antimicrobial resistance in southeast Asia reported that in Myanmar 87% of patients with upper respiratory tract infections in primary care received an antibiotic, as did 43% of patients in Thailand. A review of 32 primary care centres in northern Thailand found that 47·6% of patients with a documented fever were prescribed antibiotics.^7^, ^8^

Fever is a common reason for attending primary care facilities. Although malaria can be readily ruled out with rapid tests, the ubiquitous use of these tests as malaria declines implies that most febrile patients will have a negative test result. Health-care providers in primary care, however, have no means to diagnose other causes of acute fever, driving further inappropriate antibiotic prescription.^9^ Concurrently, patients with potentially life-threatening bacterial diseases such as scrub typhus and leptospirosis, which are widespread in southeast Asia, often receive no treatment or inappropriate antibiotics.^10^, ^11^ Ideally, pathogen-specific rapid tests would establish whether and which antibiotics are required at point of care, but use of these tests is unlikely to be feasible in the foreseeable future because of the small range of point-of-care tests that are available, with many tests being unable to distinguish between invasive infection and past exposure. Furthermore, even well resourced research studies using laboratory reference tests and paired samples rarely identify a pathogenic agent in more than half of febrile patients.^10^, ^11^, ^12^

Point-of-care tests for host-response biomarkers offer an alternative to pathogen-specific testing, with the potential for ruling out the need for antibiotic treatment and reassuring health-care providers and patients when this treatment is less likely to be required. The need for simple tests to assist in prescribing decisions has been recognised globally,^13^ but few potential biomarkers have been evaluated across a broad range of settings and populations, and none has shown perfect diagnostic performance.^14^, ^15^

**Research in context**

**Evidence before this study**

Retrospective studies have found C-reactive protein (CRP) to be highly sensitive and moderately specific in the identification of bacterial infection in blood samples from febrile patients. One such study of over 1300 microbiologically confirmed infections in patients across southeast Asia found a sensitivity of 86% and a specificity of 67% for CRP at a threshold of 20 mg/L, with an area under the curve of the receiver operating characteristic of 0·83 (95% CI 0·81–0·86). A Cochrane systematic review of six studies including more than 3000 patients in primary care who presented with acute respiratory infections in high-income settings concluded that CRP was an effective measure to reduce antibiotic prescription. Another systematic review of clinical trials of host biomarker testing for the identification of serious infections in children concluded that CRP tests could be diagnostically useful, but more evidence was needed on specific thresholds. A cluster randomised controlled trial from Belgium concluded that CRP testing should be targeted at children at risk of severe infections. Neither this study nor those in the systematic reviews originate in low-income and middle-income countries (LMICs) where the burden of infectious diseases is high and access to well trained clinicians can be low. We searched MEDLINE for studies published in English using the combination of “trial”, “fever” or “febrile”, and “C reactive protein” and identified two relevant trials from LMICs. We applied no date restriction to our search and our last search was Jan 20, 2018. In Tanzania, CRP testing was incorporated within a bundle of interventions that resulted in a large reduction in antibiotic prescribing in children attending outpatient clinics from a baseline of 94·9% to just 11·5%. In Vietnam, CRP testing alone in patients with acute respiratory tract infections reduced antibiotic prescribing from 78% in the control group to 64% in the intervention group.

**Added value of this study**

We extended the evaluation of CRP testing in southeast Asian primary care settings to all acutely febrile patients older than 12 months. The study included two intervention groups with different CRP thresholds indicating the need for antibiotics. The findings suggest that only the higher threshold of 40 mg/L was associated with significant reductions in prescribing compared with the control group, although all three study groups had significantly lower antibiotic prescription than those documented in retrospective surveys before the trial. In both intervention groups, patients with elevated CRP were more likely to be prescribed an antibiotic than those in the control group, providing an additional diagnostic safety layer that could be particularly important in settings where access to well trained clinicians is low.

**Implications of all the available evidence**

In primary care settings in southeast Asia where the prevalence of antibiotic prescription is high, CRP testing can be used to inform the management of patients with an acute fever and those with an acute respiratory tract infection.

C-reactive protein (CRP) is an acute-phase biomarker of inflammation. Although increases in CRP concentrations have a variety of causes,^16^ the utility of CRP in distinguishing between bacterial and viral infections has been shown in stored samples from febrile patients in southeast Asia across diverse settings and populations, including inpatients, outpatients, children, adults, and pregnant women.^17^, ^18^ These studies concluded that CRP is highly sensitive and moderately specific in identifying bacterial infections. Although research and development for better biomarkers continues, CRP testing is potentially a readily available means of improving prescribing decisions as a plethora of CRP point-of-care tests are commercially available,^19^ with some costing less than US$1·00.^20^ However, selecting a CRP test for use in routine care requires the identification of optimal thresholds to indicate the need for antibiotic treatment, for which scant evidence is available.

A clinical trial in Vietnamese patients with acute respiratory tract infections in primary care^21^ found that CRP testing with a threshold of 10 mg/L in children and 20 mg/L in adults reduced antibiotic prescription from 78% to 64% without altering the duration of symptoms. The objective of our clinical trial was to estimate the effect of CRP testing on antibiotic prescription in acutely febrile children and adults attending primary care in Thailand and Myanmar. Previous trials of CRP-guided antibiotic treatment used quantitative readers, which are unlikely to be available in many low-income and middle-income settings. In our trial, health-care providers were notified only as to whether CRP concentrations were low or high with respect to two proposed thresholds—20 mg/L and 40 mg/L. Identifying an optimal threshold for CRP-guided antibiotic treatment could inform the choice of lateral flow devices for use in routine primary care settings.

## Methods

### Study design and participants

This study was designed as a multicentre, open-label, randomised, controlled trial that compared CRP-guided antibiotic prescription in febrile patients with the standard prescribing practice. The design included two intervention groups with CRP thresholds of 20 mg/L and 40 mg/L to guide antibiotic prescription. These thresholds were selected on the basis of previous literature on CRP concentrations in febrile patients,^22^, ^23^ particularly studies from southeast Asia,^17^, ^18^ including a study from Chiangrai, Thailand, that evaluated 20 mg/L and 40 mg/L CRP as candidate thresholds for the identification of bacterial infections (sensitivity of 92% for 20 mg/L and 86% for 40 mg/L).^24^

In Thailand, primary care units (PCUs) in Chiangrai were selected as study sites for patient recruitment, with the intention of including facilities with high patient turnover while ensuring diversity in terms of the rural or urban environment. Two PCUs were initially included, and four additional sites were later opened because of slow recruitment. In Myanmar, the study was done in three Medical Action Myanmar (MAM) clinics and in one adjacent hospital outpatient department located in the poorest township of Yangon (table 1).^25^

**Table 1:** Trial sites

	**Chiangrai, Thailand**	**Hlaing Tha Yar, Myanmar**
Sites	Six public primary care units	Three primary care clinics and one outpatient department (government hospital)
Location	Rural and peri-urban settings within a 30 km radius of Chiangrai city centre	Slum areas and peri-urban townships on the west side of Yangon
Health-care provider	Two to three registered nurses per site	Two to five medical doctors per site
Access fees	Universal health coverage for registered citizens*	Free
Population	Thai community, 15% ethnic minorities	Mainly Burmese community
Investigations routinely available	Finger-prick blood glucose test	Rapid test for malaria
Malaria transmission	0–0·1 cases per 1000 population	0–0·1 cases per 1000 population

* THB30 (US$0·91) were previously charged per visit; this fee is now inconsistently applied.

In terms of policy environment and antimicrobial resistance awareness campaigns, Thailand has been notably active compared with other countries in the region,^7^ including through its Antibiotic Smart Use (ASU) programme, in place since 2007.^26^ The programme set a target prescription rate of 20% per month for respiratory infections and acute diarrhoea as part of the key performance indicators for PCUs; this target has been integrated in a pay-for-performance (P4P) policy of the National Health Security Office since 2009. In August, 2016 (during the study), the National Strategic Plan on antimicrobial resistance 2017–21 was endorsed by the cabinet as Thailand's first national strategy addressing antimicrobial resistance challenges, and the Ministry of Public Health (MOPH) adopted the ASU targets into its Service Plan Policy on Rational Drug Use (RDU).^27^ Unlike the previous P4P policy that provided financial incentives at the facility level, the RDU Service Plan incentivised higher-level stakeholders in the MOPH to exercise their authority in meeting the antibiotic prescription targets. To achieve this, the MOPH now relies on health inspectors to guide and encourage hospitals and PCUs to reduce the unnecessary use of antibiotics. The antimicrobial-resistance policy environment in Myanmar is not well developed, with no relevant policies introduced between 2010 and 2015.^7^

In addition to the influence of the shifting policy environment, we anticipated a potential for observation bias due to the presence of research staff and a possible contamination effect on prescribing in the control group resulting from exposure to CRP test results in the intervention groups (ie, if healthcare providers observe low frequency of patients with high CRP in the interventions groups, this might affect their prescribing in patients in the control group). Therefore, to understand the prescribing practices in febrile patients in the trial sites before intervention, we did surveys to include retrospective data from January, 2015, to December, 2016, in Thailand, and from November, 2015, to April, 2016, in Myanmar. Further details on the data collection processes for the background surveys are presented in the appendix.

All participants in the trial were aged 1 year or older with a documented fever (defined as a tympanic temperature of >37·5°C according to WHO standards) or a chief complaint of fever (<14 days), regardless of previous antibiotic intake and comorbidities other than malignancies. The exclusion criteria were as follows: infants younger than 1 year; symptoms requiring hospital referral, defined as either impaired consciousness, inability to take oral medication, or convulsions; a positive malaria test; the main complaint being trauma or injury; suspicion of either tuberculosis, urinary tract infection, or local skin or dental abscess or infection; any symptom present for more than 14 days; any bleeding; and an inability to comply with the follow-up visit at day 5. A complete list of all inclusion and exclusion criteria can be found in the protocol summary on ClinicalTrials.gov. All participants (or parents or guardians in the case of children) provided written informed consent. The protocol, informed consent form, and case record forms were reviewed and approved by the Oxford Tropical Research Ethics Committee, the Mahidol University Faculty of Tropical Medicine Ethics Committee, and the Myanmar Department of Medical Research and Chiangrai Provincial Public Health Office Research Ethics Committees.

Study staff explained the trial to potential participants and took their written informed consent to join the study before any study-specific procedures were done. In the case of participants younger than 18 years, a parent or guardian was asked to sign and date the informed consent form (ICF) and the participant was asked to give consent or assent depending on their age and local practice. In the case of illiterate patients or parents or guardians, a witness was asked to sign the ICF to confirm that the participant gave informed verbal consent to participate.

### Randomisation and masking

Individuals were randomly assigned at a 1:1:1 ratio to either one of the two intervention groups or the control group and were stratified by country (Thailand and Myanmar) and age group (children and adults, with adulthood defined as age ≥12 years). Computer-based individual randomisation was done at the Mahidol-Oxford Tropical Medicine Research Unit, Bangkok, Thailand by the trial statistician (MM) using the ralloc command in Stata version 14. Numbered, sealed, opaque envelopes containing the randomised study group were prepared by the central support team and opened sequentially on site by the study staff after patients were enrolled. Study groups consisted of intervention groups A and B, in which CRP was measured by study staff on site and the result was communicated to the health-care provider as low CRP or high CRP using cutoff thresholds of 20 mg/L (group A) or 40 mg/L (group B), and control group C, in which health-care providers were asked to manage febrile patients as per standard of care.

Health-care providers and patients were masked to allocation between the two intervention groups but by design were aware of allocation to the control group. Research staff could not be masked to patient allocation between all groups.

### Procedures

Before patient recruitment, the health-care providers were informed of the utility of CRP to help guide antibiotic prescription, while mitigating the threat of antimicrobial resistance. The information was provided by the study investigators in the local language and dialect, on the basis of previous models developed for CRP-testing-related training,^28^ and contextualised to the Thai and Myanmar settings. A refresher session was provided around the midpoint of study recruitment. Health-care providers were advised that for febrile patients with no clear danger signs and low CRP concentrations they should refrain from prescribing antibiotics, whereas for patients with high CRP the guidance was to consider prescribing antibiotics on the basis of their clinical judgment. The health-care providers were informed that the test was not of perfect accuracy in its identification of patients requiring antibiotic treatment.

Following randomisation, patients from the intervention groups had a capillary blood sample analysed for CRP on site by the study staff who used a CRP reader (NycoCard II Reader, Axis Shield, Oslo, Norway). A brief educational video on antimicrobial resistance and CRP was shown to participants in the intervention groups with the intention of ensuring patients' understanding of the test. The participants were then provided with a card specifying whether their CRP concentrations were high or low in relation to their intervention group and referred to the health-care provider. In the control group, a venous blood sample was collected by study staff, stored at 4°C, and retrospectively tested for CRP concentrations. All patients then proceeded to a routine medical examination by the primary-care health provider who decided whether an antibiotic was required. Demographic and clinical data were recorded by the study staff on a case report form (CRF).

All patients were followed up both at day 5 (allowable range 4–7 days) and day 14 (allowable range 12–16 days) after recruitment by face-to-face appointment with study staff. If patients were unable to attend a follow-up visit in person, a structured telephone interview was done instead. Patients in all three groups were tested for CRP at the second visit (day 5) on site by the study staff to help gauge clinical recovery, and health-care providers were informed if CRP concentrations were equal to or higher than 50 mg/L in children, and equal to or higher than 100 mg/L in adults.

Patients received compensation for their time and travel expenses at enrolment and on each of the follow-up visits if they reattended in person.

### Outcomes

The primary outcome was the proportion of patients who were prescribed any antibiotic at the facility from day 0 to day 5 (allowable range 4–7) in each intervention group in total, and the proportion of patients who were prescribed an antibiotic when CRP concentrations were above and below the 20 mg/L or 40 mg/L thresholds. The CRP reading was done in a central lab off-site for the control group and it was performed on site for the intervention groups. The data on prescribing were recorded independently on site and the outcome was assessed centrally. Secondary outcomes included the proportion of patients prescribed an antibiotic from day 0 to day 14 at the health facility. Clinical outcomes included patient self-reported recovery at each follow-up visit, duration and severity of symptoms, frequency of unplanned reconsultation within the 14 days of follow-up, temperature and CRP concentrations at day 5 as objective measures of clinical recovery, and occurrence of serious adverse events, defined as events requiring admission to hospital or death within 14 days of enrolment. Due to the extensive trial outputs, this manuscript reports the primary outcome and key secondary outcomes relating to antibiotic prescribing and clinical recovery. The other secondary outcomes of the study—the correlation between CRP results and clinical outcomes on day 5 of follow-up, the impact of CRP testing on antibiotic consumption obtained elsewhere after the first consultation, the attitudes and satisfaction of health-centre staff and patients towards the CRP test, the prevalence of key pathogens in febrile patients in these settings, and the ability of CRP to discriminate between viral and bacterial pathogens—will be reported elsewhere.

### Statistical analysis

We expected CRP testing to reduce antibiotic prescriptions by 25% from baseline, but with anticipated contamination between study groups, the sample size was increased to detect a reduction in antibiotic prescription by 20% independently for children and adults in each country. The sample size was adjusted further to account for multiple comparisons between the three study groups on the basis of Bonferroni's correction. An adjusted significance level (type I error) of 0·017 was used to yield a 5% overall significance level for the three comparisons. Allowing for a projected 15% loss to follow-up required 198 patients per study group, rounded to 200, to give a total of 2400 patients (600 children and 600 adults per country).

The trial was analysed by intention to treat and per protocol (appendix). The per-protocol analysis included patients for whom follow-up data were available on both day 5 and day 14, and to whom health-care providers prescribed antibiotics in accordance with test results; therefore, the effect size in the per-protocol analysis represents the potential effect of the tests under full compliance.

Differences in primary and secondary outcomes were analysed overall and in the four predefined subgroups of country and age category. Descriptive statistics for continuous variables with normal distribution used means and SD and medians with IQR for non-normally distributed continuous variables. Comparison between groups used *t* tests for normally distributed variables, the Mann-Whitney test for non-normally distributed variables, and χ^2^ test for categorical variables.

Primary and secondary outcomes between each of the intervention groups and the control group were compared using a logistic regression model; health facilities were considered to have a random effect on the primary outcome.

The difference in the number of prescriptions of various broad-spectrum antibiotics was also compared, including ceftriaxone, cefixime, ciprofloxacin, levofloxacin, azithromycin, and amoxicillin with clavulanic acid. The exhaustive list of antibiotics prescribed at the facilities is provided in the appendix. This analysis was not prespecified in the study protocol.

We generated Kaplan-Meier curves to visualise time to clinical recovery on the basis of the patient declaration, with a corresponding p value using a log-rank test for survival curves. We used a Cox regression model to quantify the difference in clinical outcomes between intervention and control groups calculating the adjusted hazard ratios (HRs), with age stratum and country as fixed effects and health-care centres as a Gaussian random effect. The 95% CIs have been provided where appropriate. Data analyses were done with Stata version 15. The Clinical Trial Support Group at the Mahidol Oxford Tropical Medicine Research Unit did two monitoring visits to each site to ensure the integrity of the data; the first visit took place after 200 children and adults were enrolled in each country, and a second at the end of the study recruitment. This trial is registered with ClinicalTrials.gov, number NCT02758821.

### Role of the funding source

The funders had no role in study design, data collection, data interpretation, or writing of the manuscript. The corresponding author (YL) had full access to all the data in the study and had the final responsibility to submit for publication.

## Results

**Figure 1:**
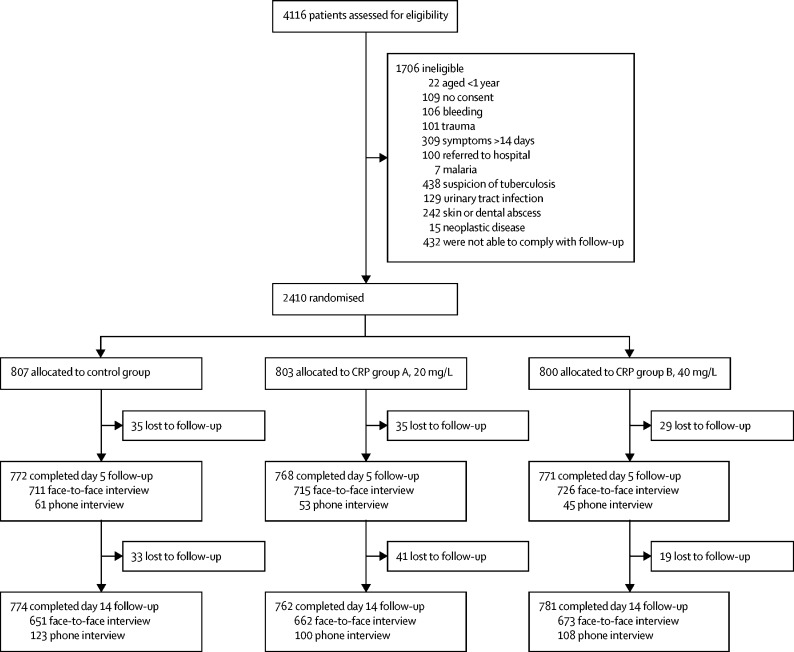
Trial profile CRP=C-reactive protein.

A total of 4116 patients were assessed for eligibility, of whom 2410 children and adults with a documented fever or a history of fever were prospectively recruited between June 8, 2016, and Aug 25, 2017, across ten facilities (figure 1). 803 patients were randomly assigned to CRP group A, 800 to CRP group B, and 807 to the control group (the intention-to-treat population). 598 patients in CRP group A, 593 in CRP group B, and 767 in the control group had follow-up data for both day 5 and day 14 and had been prescribed antibiotics in accordance with test results, so were included in the per-protocol population. Patient characteristics in the control group and the two intervention groups were similar at day 0 (table 2). Patients attended the facilities within a median of 2·5 days (IQR 1–4) after onset of symptoms. Clinically, respiratory symptoms were the most common presentation, followed by gastrointestinal and neurological symptoms (stratified results per country and age category are presented in the appendix).

**Table 2:** Day 0 characteristics comparing control group, group A (20 mg/L CRP threshold), and group B (40 mg/L CRP threshold)

		**Control group**		**CRP group A**		**CRP group B**	
		Aged <12 years (n=402)	Aged ≥12 years (n=405)	Aged <12 years (n=400)	Aged ≥12 years (n=403)	Aged <12 years (n=399)	Aged ≥12 years (n=401)
**Demographic characteristics**							
Sex							
	Male	204 (51%)	159 (39%)	209 (52%)	156 (39%)	204 (51%)	174 (43%)
	Female	198 (49%)	246 (61%)	191 (48%)	247 (61%)	195 (49%)	227 (57%)
Age, median (IQR), years		4 (2–7)	33 (22–52)	4 (2–7)	35 (20–53)	4 (2–7)	34 (21–51)
≥30 min to reach the facility		100 (25%)	66 (16%)	100 (25%)	69 (17%)	98 (25%)	81 (20%)
Presence of comorbidity*		15 (4%)	112 (28%)	16 (4%)	100 (25%)	20 (5%)	88 (22%)
Symptoms onset, median (IQR), days		2 (1–3)	3 (2–4)	2 (1–3)	3 (2–4)	2 (1–3)	3 (2–4)
Self-reported antibiotic intake		16 (4%)	25 (6%)	20 (5%)	17 (4%)	22 (6%)	29 (7%)
**Clinical characteristics and self-reported symptoms**							
Documented fever (>37·5°C)		200 (50%)	155 (38%)	203 (51%)	143 (35·5%)	223 (56%)	148 (37%)
Neurological symptoms†		62 (15%)	148 (37%)	39 (10%)	156 (39%)	40 (10%)	155 (39%)
Respiratory symptoms‡		326 (81%)	323 (80%)	315 (79%)	315 (78%)	327 (82%)	299 (75%)
Gastrointestinal tract symptoms§		104 (26%)	95 (23%)	124 (31%)	83 (21%)	109 (27%)	68 (17%)
Other symptoms¶		9 (2%)	25 (6%)	41 (10%)	37 (9%)	30 (8%)	43 (11%)

Data are number (%) or median (IQR). CRP=C-reactive protein.

* Comorbidities included HIV infection, chronic hepatitis B or C infection, cirrhosis, diabetes, asthma, anaemia, chronic obstructive pulmonary disease, gastritis, congenital heart or kidney disease, alcoholism, dyslipidaemia, glucose-6-phosphate dehydrogenase deficiency, hypertension, rheumatic heart disease, thalassaemia, or thyroid disease.

† Neurological symptoms include headache, confusion, dizziness, or hearing loss.

‡ Respiratory symptoms include sore throat, dyspnoea, chest pain, runny nose, or cough.

§ Gastrointestinal symptoms include nausea, vomiting, diarrhoea, or abdominal pain.

¶ Other symptoms declared were defined by the presence of fever alone or symptoms other than those present in neurological, respiratory, or gastrointestinal symptoms. Common symptoms in this group included myalgia, arthralgia, jaundice, tiredness, chills, sweating, weight loss, skin eruption, dysuria, or eye redness.

**Figure 2:**
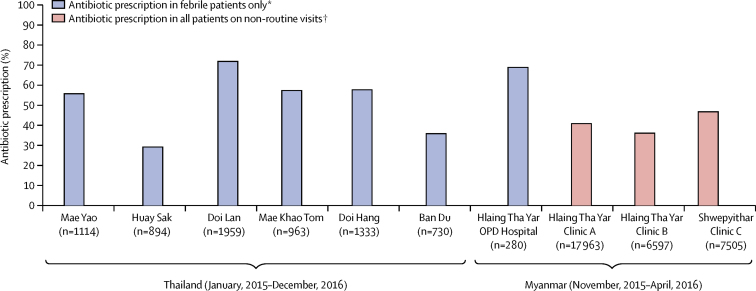
Background antibiotic prescription in Thailand and Myanmar OPD=outpatient department. *Estimated prescriptions in patients for whom clinical data on febrile status were available. †Estimated prescriptions in all patients on non-routine visits (febrile status unknown).

The retrospective background surveys in the six Thai sites (6993 patients in total) showed that between 260 (29%) of 894 patients and 1406 (72%) of 1959 patients with a history of fever or a documented fever were prescribed antibiotics (figure 2). In patients with a documented fever, between 123 (37%) of 330 patients and 453 (87%) of 518 patients in each of the sites were prescribed an antibiotic; these proportions were largely unchanged in the 2 years preceding the study as was generally the case in a review of prescribing practices in all PCUs in the same district.^8^ Among the four Myanmar sites (32 345 patients in total), only the Hlaing Tha Yar government hospital outpatient department had patient records that included febrile status and antibiotic prescription, in which 173 (69%) of 252 patients with a documented fever were prescribed an antibiotic. In the three MAM clinics, data were only available on the total number of non-routine visits (ie, excluding patients attending the clinic for HIV care, tuberculosis care, antenatal appointments, and family planning, as well as malnourished children), without a record of febrile status, and only the overall number of antibiotics prescribed during the corresponding period was known. Together, the pooled absolute numbers indicate that approximately 41% of non-routine clinic attendees received an antibiotic.

In the intention-to-treat population, we observed a significant difference in the trial primary outcome of antibiotic prescription from day 0 up to day 5 between the control group (318 [39%] of 807) and patients in group B (275 [34%] of 800), with a risk difference of −5·0 percentage points (95% CI −9·7 to −0·3) and an adjusted odds ratio (aOR) of 0·80 (95% CI 0·65 to 0·98; table 3). In group A, 290 (36%) of 803 patients were prescribed an antibiotic from day 0 to day 5, a non-significant difference compared with control group (risk difference −3·3 percentage points, 95% CI −8·0 to 1·4; aOR 0·86, 95% CI 0·70 to 1·06). The per-protocol analysis (appendix) showed a −20·4 percentage point difference (95% CI −25 to −15·7) in antibiotic prescription for the primary outcome in group B compared with the control group (aOR 0·35, 95% CI 0·27 to 0·45) and a −12·3 percentage point difference (–17·3 to −7·4) in group A compared with the control group (aOR 0·56, 0·44 to 0·71).

**Table 3:** Antibiotic prescription in the control group, group A (20 mg/L CRP threshold), and group B (40 mg/L CRP threshold)

	**Control group**	**CRP group A**	**Risk difference (95% CI)**	aOR*(95% CI)	**CRP group B**	**Risk difference (95% CI)**	aOR*(95% CI)
**All age groups in Thailand and Myanmar**							
Number of participants	807	803			800		
On day 0	297 (36·8%)	269 (33·5%)	−3·3 (−8·0 to 1·4)	0·86 (0·70 to 1·06)	245 (30·6%)	−6·2 (−10·8 to −1·6)	0·75 (0·60 to 0·92)
Between day 0 and day 5	318 (39·4%)	290 (36·1%)	−3·3 (−8·0 to 1·4)	0·86 (0·70 to 1·06)	275 (34·4%)	−5·0 (−9·7 to −0·3)	0·80 (0·65 to 0·98)
Between day 0 and day 14	323 (40·0%)	292 (36·4%)	−3·7 (−8·4 to 1·1)	0·85 (0·69 to 1·04)	279 (34·9%)	−5·2 (−10·0 to −0·4)	0·79 (0·64 to 0·98)
**Patients aged <12 years in Thailand**							
Number of participants	195	194			193		
On day 0	64 (32·8%)	56 (28·9%)	−4·0 (−13·1 to 5·2)	0·83 (0·53 to 1·28)	49 (25·4%)	−7·4 (−16·4 to 1·6)	0·68 (0·43 to 1·08)
Between day 0 and day 5	68 (34·9%)	61 (31·4%)	−3·4 (−12·8 to 5·9)	0·85 (0·55 to 1·31)	52 (26·9%)	−7·9 (−17·1 to 1·2)	0·68 (0·43 to 1·06)
Between day 0 and day 14	69 (35·4%)	61 (31·4%)	−3·9 (−13·3 to 5·4)	0·83 (0·54 to 1·28)	52 (26·9%)	−8·4 (−17·6 to 0·7)	0·66 (0·42 to 1·03)
**Patients aged ≥12 years in Thailand**							
Number of participants	201	200			199		
On day 0	63 (31·3%)	57 (28·5%)	−2·8 (−11·8 to 6·1)	0·86 (0·56 to 1·34)	65 (32·7%)	1·3 (−7·8 to 10·5)	1·06 (0·69 to 1·63)
Between day 0 and day 5	64 (31·8%)	60 (30·0%)	−1·8 (−10·9 to 7·2)	0·91 (0·59 to 1·40)	68 (34·2%)	2·3 (−6·9 to 11·5)	1·12 (0·73 to 1·71)
Between day 0 and day 14	64 (31·8%)	60 (30·0%)	−1·8 (−10·9 to 7·2)	0·91 (0·59 to 1·40)	69 (34·7%)	2·8 (−6·4 to 12·1)	1·14 (0·74 to 1·75)
**Patients aged <12 years in Myanmar**							
Number of participants	207	206			206		
On day 0	78 (37·7%)	77 (37·4%)	−0·3 (−9·6 to 9·0)	0·99 (0·66 to 1·48)	65 (31·6%)	−6·1 (−15·3 to 3·0)	0·76 (0·50 to 1·15)
Between day 0 and day 5	87 (42·0%)	84 (40·8%)	−1·3 (−10·8 to 8·3)	0·95 (0·64 to 1·41)	79 (38·4%)	−3·7 (−13·1 to 5·8)	0·86 (0·57 to 1·29)
Between day 0 and day 14	88 (42·5%)	86 (41·8%)	−0·8 (−10·3 to 8·8)	0·97 (0·66 to 1·44)	82 (39·8%)	−2·7 (−12·2 to 6·8)	0·90 (0·60 to 1·34)
**Patients aged ≥12 years in Myanmar**							
Number of participants	204	203			202		
On day 0	92 (45·1%)	79 (38·9%)	−6·2 (−15·8 to 3·4)	0·78 (0·52 to 1·15)	66 (32·7%)	−12·4 (−21·8 to −3·0)	0·58 (0·38 to 0·87)
Between day 0 and day 5	99 (48·5%)	85 (41·9%)	−6·7 (−16·3 to 2·3)	0·76 (0·52 to 1·13)	76 (37·6%)	−10·9 (−20·5 to −1·3)	0·63 (0·42 to 0·94)
Between day 0 and day 14	102 (50·0%)	85 (41·9%)	−8·1 (−17·8 to 1·5)	0·72 (0·49 to 1·07)	76 (37·6%)	−12·4 (−22·0 to −2·8)	0·59 (0·40 to 0·89)

The prescription of antibiotics from day 0 to day 5 is the primary outcome. Unadjusted odds ratios are presented in the appendix. Data are number or number (%) unless otherwise stated. aOR=adjusted odds ratio. CRP=C-reactive protein.

* aORs were adjusted by site as a random effect.

**Figure 3:**
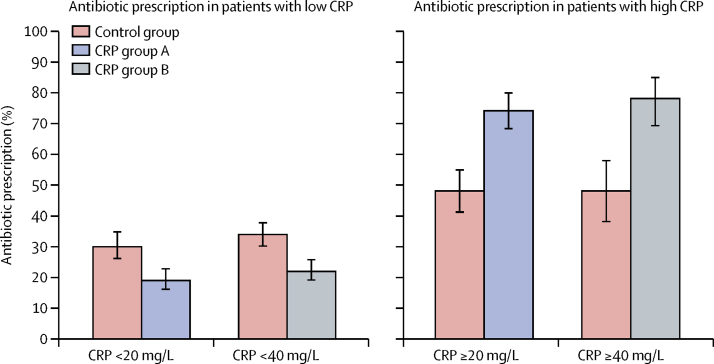
Antibiotic prescription on day 0 in relation to the CRP thresholds in each of the intervention groups for all age categories and countries Error bars represent 95% CI. CRP=C-reactive protein.

The trial primary outcome also included antibiotic prescription in relation to the CRP thresholds, with a higher proportion of patients with elevated CRP concentrations being prescribed an antibiotic in the intervention groups than in the control group (CRP ≥20 mg/L: 153 [74%] of 206 patients in group A *vs* 103 [48%] of 214 in the control group, p<0·0001; CRP ≥40 mg/L: 92 [78%] of 118 patients in group B *vs* 51 [48%] of 107 in the control group, p<0·0001; figure 3). Conversely, in patients with low CRP concentrations, antibiotic prescription was lower in the intervention groups than in the control group (CRP <20 mg/L: 119 [20%] of 595 patients in group A *vs* 134 [30%] of 445 in the control group, p<0·0001; CRP <40 mg/L: 153 [22%] of 682 patients in group B *vs* 186 [34%] of 552 in the control group, p<0·0001). Considering a threshold of 20 mg/L CRP as indicative of requiring antibiotics, 414 (63%) of 659 patients in the control group had an antibiotic correctly prescribed or withheld, as compared with 632 (79%) of 801 in group A. Similarly, assuming a threshold of 40 mg/L as indicative of the need for an antibiotic, 417 (63%) of 659 in the control group had an antibiotic appropriately prescribed or withheld, as compared with 621 (78%) of 800 in group B. Therefore, compliance with the test result was similarly high in the two intervention groups.

The risk difference in prescription in the subgroup of patients presenting with a documented fever was −7·5 percentage points (95% CI −14·7 to −0·3) in group B compared with the control group, and −3·7 percentage points (–11·0 to 3·7) in group A compared with the control group (table 4). Patients presenting with a respiratory syndrome in both intervention groups showed a significant reduction in antibiotic prescription, but did not show a significant reduction in the subgroup of patients with a neurological syndrome was observed. For patients with gastrointestinal symptoms, we did not observe a significant increase in prescription in the intervention groups compared with the control group.

**Table 4:** Subgroup analysis for antibiotic prescription

	**Control group (n=807)**	**CRP group A (n=803)**	**Risk difference (95% CI)**	aOR*(95% CI)	**CRP group B (n=800)**	**Risk difference (95% CI)**	aOR*(95% CI)
**Neurological presentation**							
Number of participants	210	195	..	..	195	..	..
On day 0	83 (40%)	71 (36%)	−3·1% (−12·6 to 6·3)	0·85 (0·56 to 1·29)	65 (33%)	−6·2% (−15·6 to 3·2)	0·71 (0·46 to 1·10)
Between day 0 and day 5	90 (43%)	78 (40%)	−2·9% (−12·5 to 6·7)	0·87 (0·58 to 1·31)	69 (35%)	−7·5% (−17·0 to 2·0)	0·68 (0·45 to 1·05)
Between day 0 and day 14	92 (44%)	78 (40%)	−3·8% (−13·4 to 5·8)	0·84 (0·55 to 1·26)	69 (35%)	−8·4% (−17·9 to 1·1)	0·65 (0·43 to 1·00)
**Respiratory presentation**							
Number of participants	649	630	..	..	626	..	..
On day 0	263 (41%)	218 (35%)	−5·9% (−11·2 to −0·6)	0·79 (0·62 to 0·99)	192 (31%)	−9·9% (−15·1 to −4·6)	0·63 (0·50 to 0·81)
Between day 0 and day 5	281 (43%)	237 (38%)	−5·7% (−11·1 to −0·3)	0·80 (0·63 to 1·00)	221 (35 %)	−8·0% (−13·3 to −2·7)	0·70 (0·55 to 0·89)
Between day 0 and day 14	284 (44%)	238 (38%)	−6·0% (−11·4 to −0·6)	0·79 (0·62 to 0·99)	225 (36%)	−7·8% (−13·2 to −2·5)	0·71 (0·56 to 0·90)
**Gastrointestinal presentation**							
Number of participants	199	207	..	..	177	..	..
On day 0	65 (33%)	75 (36%)	3·6% (−5·7 to 12·8)	1·17 (0·78 to 1·77)	63 (36%)	2·9% (−6·7 to 12·5)	1·16 (0·75 to 1·80)
Between day 0 and day 5	74 (37%)	82 (40%)	2·4% (−7·0 to 11·9)	1·11 (0·74 to 1·66)	67 (38%)	0·6% (−9·1 to 10·5)	1·04 (0·68 to 1·60)
Between day 0 and day 14	74 (37%)	83 (40%)	2·9% (−6·6 to 12·4)	1·13 (0·76 to 1·69)	67 (38%)	0·7% (−1·1 to 10·5)	1·04 (0·68 to 1·60)
**Documented fever**							
Number of participants	355	346	..	..	371	..	..
On day 0	165 (47%)	148 (41%)	−3·7% (−11·1 to 3·7)	0·85 (0·63 to 1·15)	141 (38%)	−8·5% (−15·8 to −1·3)	0·66 (0·49 to 0·90)
Between day 0 and day 5	173 (49%)	156 (45%)	−3·7% (−11·0 to 3·7)	0·85 (0·63 to 1·15)	153 (41%)	−7·5% (−14·7 to −0·3)	0·71 (0·52 to 0·96)
Between day 0 and day 14	175 (49%)	157 (45%)	−3·9% (−11·3 to 3·5)	0·84 (0·62 to 1·14)	154 (41%)	−7·8% (−15·0 to −0·6)	0·70 (0·51 to 0·95)

Data are number or number (%) unless otherwise stated. The prescription of antibiotics from day 0 to day 5 is the primary outcome. aOR=adjusted odds ratio. CRP=C-reactive protein.

* aORs were adjusted by site as a random effect.

Considering the follow-up period between day 0 and day 14, antibiotic prescription was −3·7 percentage points (95% CI −8·4 to 1·1) in group A, and −5·2 percentage points (95% CI −10·0 to −0·4) in group B, compared with the control group (table 3).

A post-hoc analysis showed that the prescription of broad-spectrum antibiotics (appendix) was lower at enrolment and throughout the follow-up visits in group B than in the control group (aOR 0·44, 95% CI 0·26–0·72 at day 0; aOR 0·79, 95% CI 0·64–0·97 from day 0 to day 5; and aOR 0·79, 95% CI 0·64–0·98 from day 0 to day 14), whereas this reduction was only significant on day 0 in group A (aOR 0·60, 95% CI 0·38–0·96).

**Figure 4:**
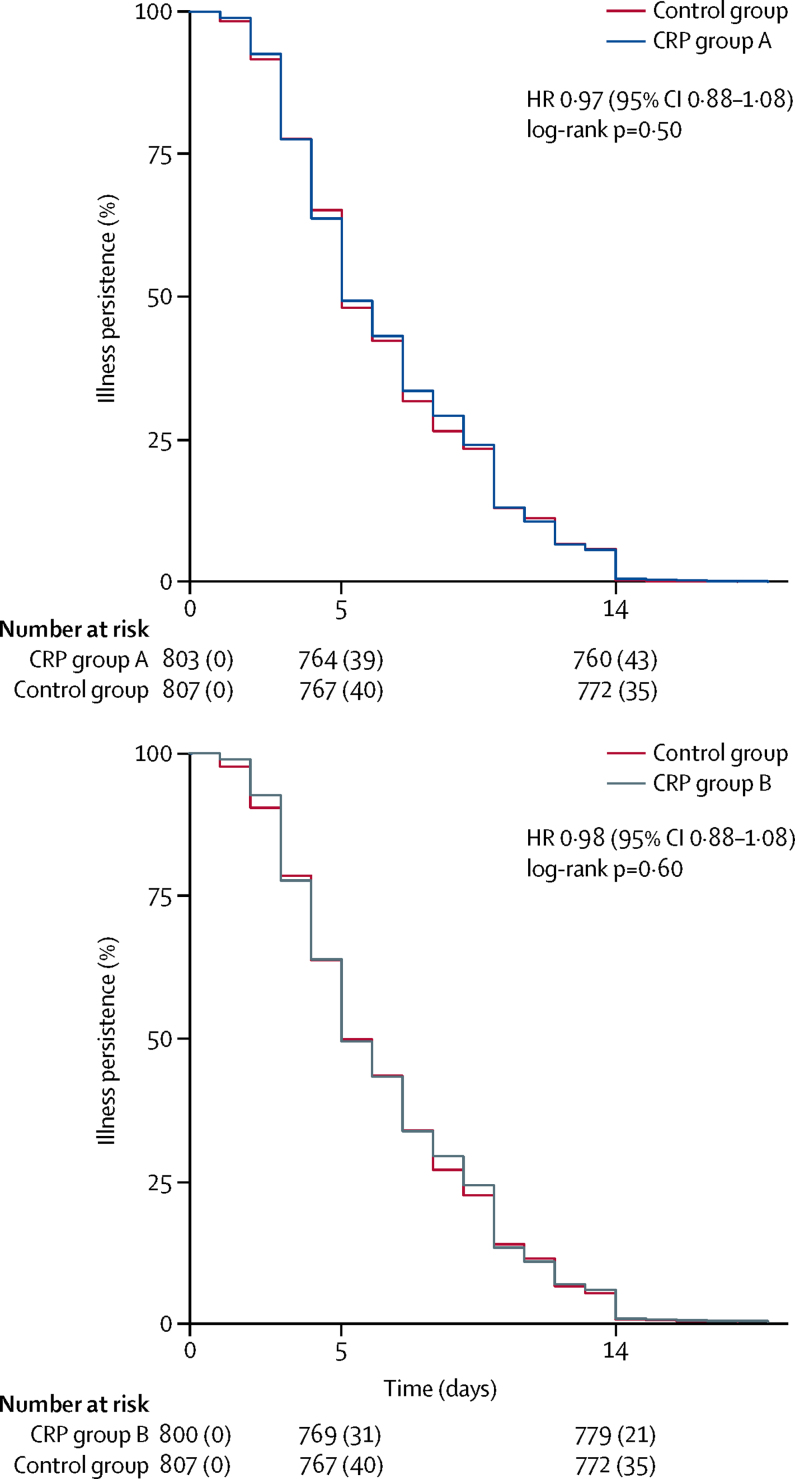
Kaplan-Meier curves of symptom duration in the control group versus group A (20 mg/L) and group B (40 mg/L)

We observed no differences in the prevalence of recovery as defined by patient declaration at day 5 or day 14 of follow-up between the study groups, with 495 (65%) of 764 at day 5 and 718 (94%) of 760 at day 14 in the 20 mg/L CRP group reporting recovery compared with 488 (63%) of 769 at day 5 and 733 (94%) of 779 at day 14 in the 40 mg/L CRP group, and 491 (64%) of 767 at day 5 and 738 (96%) of 772 at day 14 in the control group (appendix). We present a comparison of the time-to-event curves for recovery between groups A and B and the control group (figure 4). The corresponding log-rank test and HRs in group A and group B were non-significant. Similarly, no differences were found in these outcomes in the per-protocol analysis (appendix).

In cases of persistent illness at day 5 and day 14 of follow-up, the median symptom severity score corresponded to the mildest grade (ie, a severity score of 1 in a scale ranging from 1 to 4) and did not differ across the groups (appendix). The proportion of patients who attended unscheduled visits was 16 (2%) of 807 in the control group, 13 (2%) of 803 in group A, and 22 (3%) of 800 in group B with no significant difference between groups. CRP on day 5 was sufficiently elevated above the predefined threshold to notify health-care providers (50 mg/L in children and 100 mg/L in adults) in eight (1%) of 706 patients in the control group, eight (1%) of 706 in CRP group A, and six (1%) of 726 in CRP group B with no significant difference between study groups. 74 (3%) of 2150 patients had a tympanic temperature of more than 37·5°C on day 5 and 31 (2%) of 1951 on day 14 with no significant differences between the study groups. Further details on clinical outcomes by age and country are presented in the appendix.

Among the 2410 study participants, 24 severe adverse events occurred (23 hospital admissions and one death), with ten (1%) severe adverse events in group A, 11 (1%) in group B, and three (<1%) in the control group (p=0·064 for group A and p=0·043 for group B compared with the control group). Among these 24 severe adverse events, 23 were classified as being not related to the study (nine in group A, 11 in group B, and three in the control group), and one was classified as being possibly related, occurring in a woman aged 25 years who had an abdominal complaint and fever with low CRP (randomly assigned to group A), initially diagnosed by the attending health-care provider as a hypersensitivity reaction, and no antibiotic was prescribed. During admission the woman was diagnosed with mesenteric lymphadenitis, and was prescribed antibiotics and discharged alive. No microbiological investigations were done at the hospital. One death occurred during the study, which was that of a man aged 78 years who was randomly assigned to group A and had chronic obstructive pulmonary disease and heart disease. The man's CRP concentration was high, and antibiotics were prescribed on day 0; this severe adverse event was classified as not related to the study.

## Discussion

In this trial we have shown that, in primary care in southeast Asia, CRP testing with a threshold of 40 mg/L reduced antibiotic prescription in patients with a fever with no evidence of a difference in clinical recovery. Reductions in prescription were greater in patients with a documented fever, in those with respiratory presentation, and for broad-spectrum antibiotics. In addition to reducing antibiotic prescription, patients in the intervention groups with high CRP concentrations were more likely to be prescribed an antibiotic and those with low CRP concentrations were more likely to have antibiotics withheld than patients in the control group. These effects were more pronounced within group B, in which a higher threshold of 40 mg/L was used, whereas the differences between group A and the control group were of lesser magnitude and mostly not of statistical significance.

The effect size was smaller than anticipated when comparing the intervention and control groups but the difference in the proportion of patients who were prescribed antibiotics seemed to be much larger when comparing our trial data with our retrospective 2015–16 antibiotic prescription data; whether these reductions in prescribing, small or large, can have a real effect on mitigating drug pressure and the development of antimicrobial resistance is difficult to establish. With the majority of human antibiotic consumption occurring in the community and in patients with fevers and respiratory illness in particular, even small reductions in prescription could imply a large alleviation of drug pressure. Further modelling and cost-effectiveness analyses are required to explore whether these reductions and the cost of achieving them are warranted from an economic and global health perspective. A modelling analysis conservatively estimated that in the Thai context the economic costs of antimicrobial resistance per course of broad-spectrum penicillins (widely used in this study) was approximately $10. A reduction of just 10 percentage points from baseline with the use of a CRP test costing $1 could therefore be considered cost beneficial from a societal perspective.^29^

The risk of serious bacterial infection in patients in primary care without clear clinical danger signs is low, and the majority of patients in the trial had low CRP concentrations on day 0 (<20 mg/L in 72% of patients and <40 mg/L in 86%; appendix). As CRP is known to be of high sensitivity but only moderate specificity in the detection of bacterial infections, the higher threshold of 40 mg/L is likely to be appropriate for these settings, and stricter adherence to test results at this threshold would generate larger reductions in prescribing, as shown in the per-protocol analysis (>20% reduction for the primary outcome between group B and the control group; appendix). This reduction in prescribing might be achieved in scale-up programmes driven by local health authorities supported by high-level policy change. Integrating CRP testing into clinical guidelines could also enhance prescribers' confidence, as might electronic user-friendly algorithms that strengthen the consistency of fever management.^30^ Clinical and communication skills training could also enhance compliance with these algorithms.^31^

A systematic review of studies on biomarker testing in febrile children in emergency departments, all in high-income settings, concluded that CRP testing could be diagnostically useful, recommending a low threshold of 5 mg/L to rule out serious infection and a threshold of 80 mg/L to suggest the presence of serious infection.^32^ A cluster randomised trial in Belgium concluded that CRP testing in primary care should be targeted at children at high risk of severe infection after clinical assessment.^33^ This targeting could also avoid medicalisation of minor ailments. A multicountry European study showed enhanced pneumonia prediction in patients in primary care presenting with acute cough when CRP testing was added to a clinical algorithm based on symptoms alone.^34^ This risk stratification relied on the availability of experienced clinicians. In Thailand, which has a well-developed public health sector, such strategies could be relevant, with adequate supervision of health-care providers' clinical skills combined with clear referral and follow-up systems to ensure patient safety. The low number of prescriptions in the Thai control group suggests that reductions in prescribing could be attained with better training, supervision, or financial incentives, with CRP testing offering modest additional gains.

The evidence base for the evaluation of the effect of CRP-guided treatment from lower-income settings is small, and, because of particular comorbidities and pathogen exposure in lower-income countries in the tropics, data from high-income (non-tropical) settings are not easily comparable. A Tanzanian-based clinical trial on fever management showed that a host of interventions including CRP and procalcitonin tests, pulse oximetry, haemoglobin tests, and an electronic patient management algorithm, were able in combination to reduce prescribing from 95% to approximately 10% of patients.^30^ The costs and benefits of implementing a more comprehensive bundle of interventions need to be weighed against the lower costs and benefits of CRP testing alone. A trial using only CRP tests in acute respiratory tract infections in primary care in Vietnam reported a 20% reduction in prescribing on first attendance.^21^

Data for the retrospective survey in Thailand were obtained from a search of routinely collected electronic medical records, making the verification of the data challenging; however, CRF data from a subsample of patients included in this retrospective survey were compared with their respective routine medical records and were found to be consistent.^8^ The retrospectively collected 2015–16 antibiotic prescription data for the MAM sites did not include patients' febrile status and only had the total number of prescriptions in the corresponding months, therefore our retrospectively collected prescription data are likely to underestimate the actual proportion of patients with a suspected infection who were prescribed an antibiotic. Despite these limitations, the retrospective surveys indicate much higher prescription rates than those observed in the research environment.

The primary outcome of the study, antibiotic prescription in response to CRP testing, is behavioural rather than biological, and therefore results need careful interpretation that considers contextual factors such as the broader policy environment and study design biases. In Thailand, reductions in prescribing were probably driven by policy changes implemented during the study period. Although our study adopted a mixed-methods approach that included social research components to better understand these contextual factors,^35^ disentangling and quantifying their relative contribution is challenging. The addition of a diagnostic tool did, however, enable further reductions in prescribing, supporting a multifaceted approach.

The reduction in prescribing in all study groups could also be explained by the Hawthorne effect because of the presence of research staff on site who were carrying out the CRP tests and monitoring prescribing. Prescribing in the control group might also have been affected by contamination (because of recognition that most patients have low CRP concentrations and do not require an antibiotic), therefore underestimating the effect that CRP testing could have in a routine care environment. The ideal study design would therefore have been a cluster randomised trial without the presence of research staff on site and with the tests being performed by routine health-care providers.

Our study was powered to detect a difference in antibiotic prescription, rather than clinical outcomes. The reason we chose this endpoint was that CRP is already widely used in hospital settings in the management of febrile patients^32^ and has been shown to be highly sensitive in detecting bacterial infections in the region.^18^ In the context of primary care and with the exclusion of patients presenting with clear danger signs, we expected very few severe outcomes, as was indeed the case. Although powering the study to detect differences in such rare events would not be feasible, the study recruited more than 2400 patients with high follow-up and no evidence of a difference between the groups was detected in the extensive measures of clinical outcome. Furthermore, in our study CRP tests were provided as an aid to health-care providers' clinical judgment, with only soft recommendations on how the tests could inform their prescribing decisions.

CRP testing in a southeast Asian primary care setting has previously been shown to reduce antibiotic prescribing in patients with respiratory tract infection and our trial extends this evidence base to those with a fever. Together, these patients represent the majority of acute primary care attendees. The fact that the proportion of patients in the control group who were prescribed an antibiotic seemed to be lower than our retrospectively collected data on antibiotic prescribing in the region indicates that substantial progress is attainable through other mechanisms, with CRP testing using a threshold of 40 mg/L bringing a modest incremental effect. These findings correspond with previous studies recommending a restricted use of CRP tests in high-risk groups, including children with high fever. This restricted use might well be viable in facilities staffed by experienced clinicians and where patient follow-up is feasible. In more peripheral settings, with minimally trained health-care providers and where follow-up of patients is a challenge, testing all febrile patients could provide an additional safety net, ensuring that patients requiring treatment are identified as such. In these settings, low-cost lateral flow devices are most likely to be relevant, for which we provide further evidence on the optimal threshold. Introduction of the tests should be planned in consideration of the local policy environment and accompanied by training for health-care providers and education for patients on the ability of CRP testing to identify when antibiotic treatment is required, and the need for better targeting of antibiotics in the fight against antimicrobial resistance.
