# Supplementary material for: Effect of point-of-care C-reactive protein testing on antibiotic prescription in febrile patients attending primary care in Thailand and Myanmar: an open-label, randomised, controlled trial
Source: Lancet Glob Health. 2018 Dec 13;7(1):e119–31. doi: 10.1016/S2214-109X(18)30444-3 (PMC6293968; doi:10.1016/S2214-109X(18)30444-3)
Supplement: Supplementary appendix [file mmc1.pdf]

THE LANCET
Global Health

Supplementary appendix

This appendix formed part of the original submission and has been peer reviewed. We post it as supplied by the authors.

Supplement to: Althaus T, Greer RC, Swe MMM, et al. Effect of point-of-care C-reactive protein testing on antibiotic prescription in febrile patients attending primary care in Thailand and Myanmar: an open-label, randomised, controlled trial. *Lancet Glob Health* 2019; **7:** e119–31.

Randomised Controlled Trial of Point-of-Care C-Reactive Protein Testing on Antibiotic Prescription in Febrile Patients Attending Primary Care in South-East Asia

Supplementary material

Background survey data collection

Thailand:

A retrospective survey was carried out on routinely collected medical records from patients attending primary care facilities with a history of fever, documented temperature > 37.5°C, ICD 10 code for infection or those prescribed an antibiotic. Data were collected via a computerised search of patient records from the primary care facilities in Chiangrai District between January 2015 and December 2016. The data presented here include only the six sites included in the trial up to the beginning of enrolment (if this occurred prior to December 2016). With the approval of the Chiangrai Provincial and Public Health Office (PHO), a research data manager accessed the PHO's routine medical records database to search for relevant patients and extract the pre-specified variables.

Myanmar:

Data on antibiotics are routinely recorded in hard copy ("Drug book") by the facility staff based in the drug storage area in each site, controlling the drug delivery to the patient according to the healthcare worker prescription. This book is stored in a locked room outside of clinic hours. Monthly stock assessments ensure the consistency between drug delivery and remaining stocks. The "Drug book" records each patient's prescription including antibiotic type and dose, but does not record the cause of the consultation, making the estimate of antibiotic prescription rate among febrile patients impossible. Among the four Myanmar sites, only the government hospital OPD had patient records that included clinical diagnosis including febrile status and antibiotic prescription, while in the three MAM clinics data were only available on the total number of non-routine visits (i.e. excluding those attending for HIV and TB care, antenatal clinic, family planning and malnourished children), without clinical diagnoses or febrile status, and only the overall number of antibiotics prescribed during the corresponding period was known.

The background data collection was approved as a part of the study protocol by the Department of Medical Research (DMR) Ethical Review Committee.

Table 1. Day 0 characteristics comparing controls and combined CRP groups per country and age category (adults defined as ≥12 years of age)

Day 0 characteristics	Thailand children		Thailand Adults		Myanmar Children		Myanmar adults	
	Controls (n=195)	CRP groups (n=387)	Controls (n=201)	CRP groups (n=399)	Controls (n=207)	CRP groups (n=412)	Controls (n=204)	CRP groups (n=405)
**Demographic characteristics**								
Male, n (%)	101 (51.8)	206 (53.2)	83 (41.3)	162 (40.6)	103 (49.8)	207 (50.2)	76 (37.3)	168 (41.5)
Age, median (IQR)	6 (3-9)	4 (3-7)	46 (26-58)	44 (23-56)	3 (2-6)	4 (2-6)	29 (19-42)	28 (20-43)
Percentage of children<5-year-old, n (%)	3 (2-4)	3 (2-4)	NA	NA	2 (1-3)	2 (1-3)	NA	NA
Presence of comorbidity, n (%)	8 (4.1)	22 (5.7)	49 (24.4)	92 (23.1)	7 (3.4)	14 (3.4)	63 (30.9)	96 (23.7)
Symptom onset (in days), median (IQR)	2 (1-3)	2 (1-3)	2 (2-3)	2 (2-3)	2 (2-3)	3 (2-3)	3 (2-5)	3 (2-5)
≥ 30min to reach the facility, n (%)	45 (23.1)	78 (20.2)	16 (8.0)	29 (7.3)	55 (26.6)	120 (29.1)	50 (24.5)	121 (29.9)
Self-reported antibiotic intake, n (%)	9 (4.6)	16 (4.1)	18 (9.0)	25 (6.3)	7 (3.4)	26 (6.3)	7 (3.4)	21 (5.2)
**Clinical characteristics and self-reported symptoms**								
Documented fever (>37.5˚C), n (%)	69 (35.4)	147 (38.0)	51 (25.4)	107 (26.8)	131 (63.3)	279 (67.7)	104 (51.0)	183 (45.2)
Neurological symptoms, n (%)	51 (26.2)	60 (15.5)	85 (42.3)	178 (44.6)	11 (5.3)	19 (4.6)	63 (30.9)	133 (32.8)
Respiratory symptoms, n (%)	175 (89.7)	343 (88.6)	178 (88.6)	351 (88.0)	151 (73.0)	299 (72.6)	145 (71.1)	263 (64.9)
Gastrointestinal symptoms, n (%)	41 (21.0)	89 (23.0)	46 (22.9)	56 (14.0)	63 (30.4)	144 (35.0)	49 (24.1)	95 (23.5)
Other symptoms, n (%)	10 (5.1)	30 (7.8)	8 (4.0)	22 (5.5)	26 (12.6)	43 (10.4)	15 (7.4)	62 (15.3)

Comorbidities included HIV, chronic hepatitis B or C, cirrhosis, diabetes mellitus, asthma, chronic anaemia, chronic obstructive pulmonary disease, chronic gastritis, congenital heart or kidney disease, chronic alcoholism, dyslipidaemia, G6PD deficiency, hypertension, rheumatic heart disease, thalassaemia, thyroid disease.
Neurological symptoms include headache, confusion, dizziness or hearing loss.
Respiratory symptoms include sore throat, dyspnoea, chest pain, runny nose, or cough.
Gastrointestinal symptoms include nausea, vomiting, diarrhoea, or abdominal pain.
Other symptoms declared were defined by the presence of fever alone or symptoms other than those present in neurological, respiratory, nor gastrointestinal symptoms. Common symptoms in this group included myalgia, arthralgia, jaundice, tiredness, chills, sweating, weight loss, skin eruption, dysuria, or eye redness.

Antibiotic prescription

Table 2. Antibiotic prescription in the controls, Group A (20mg/L) and Group B (40mg/L). Unadjusted (OR) and adjusted odds ratios (aOR) compare intervention groups with the controls.
*aOR were adjusted by site as a random effect

	Controls (n=807)	Group A (n=803)	OR (95% CI)	aOR* (95% CI)	Group B (n=800)	OR (95% CI)	aOR* (95% CI)
Overall (Children & Adults - Thailand & Myanmar)							
On Day 0, n (%)	297 (36.8)	269 (33.5)	0.87 (0.71, 1.06)	0.86 (0.70, 1.06)	245 (30.6)	0.76 (0.62, 0.93)	0.75 (0.60, 0.92)
Between Day 0 - Day 5, n (%)	318 (39.4)	290 (36.1)	0.87 (0.71, 1.06)	0.86 (0.70, 1.06)	275 (34.4)	0.81 (0.66, 0.99)	0.80 (0.65, 0.98)
Between Day 0 - Day 14, n (%)	323 (40.0)	292 (36.4)	0.86 (0.70, 1.05)	0.85 (0.69, 1.04)	279 (34.9)	0.80 (0.66, 0.98)	0.79 (0.64, 0.98)
Thailand - children	*n=195*	*n=194*			*n=193*		
On Day 0, n (%)	64 (32.8)	56 (28.9)	0.83 (0.54, 1.28)	0.83 (0.53, 1.28)	49 (25.4)	0.70 (0.45, 1.08)	0.68 (0.43, 1.08)
Between Day 0 - Day 5, n (%)	68 (34.9)	61 (31.4)	0.86 (0.56, 1.31)	0.85 (0.55, 1.31)	52 (26.9)	0.69 (0.45, 1.06)	0.68 (0.43, 1.06)
Between Day 0 - Day 14, n (%)	69 (35.4)	61 (31.4)	0.84 (0.55, 1.28)	0.83 (0.54, 1.28)	52 (26.9)	0.67 (0.44, 1.04)	0.66 (0.42, 1.03)
Thailand - adults	*n=201*	*n=200*			*n=199*		
On Day 0	63 (31.3)	57 (28.5)	0.87 (0.57, 1.34)	0.86 (0.56, 1.34)	65 (32.7)	1.06 (0.70, 1.62)	1.06 (0.69, 1.63)
Between Day 0 - Day 5, n (%)	64 (31.8)	60 (30.0)	0.92 (0.60, 1.40)	0.91 (0.59, 1.40)	68 (34.2)	1.11 (0.73, 1.69)	1.12 (0.73, 1.71)
Between Day 0 - Day 14, n (%)	64 (31.8)	60 (30.0)	0.92 (0.60, 1.40)	0.91 (0.59, 1.40)	69 (34.7)	1.14 (0.75, 1.72)	1.14 (0.74, 1.75)
Myanmar - children	*n=207*	*n=206*			*n=206*		
On Day 0, n (%)	78 (37.7)	77 (37.4)	0.99 (0.66, 1.47)	0.99 (0.66, 1.48)	65 (31.6)	0.76 (0.51, 1.15)	0.76 (0.50, 1.15)
Between Day 0 - Day 5, n (%)	87 (42.0)	84 (40.8)	0.95 (0.64, 1.41)	0.95 (0.64, 1.41)	79 (38.4)	0.86 (0.58, 1.27)	0.86 (0.57, 1.29)
Between Day 0 - Day 14, n (%)	88 (42.5)	86 (41.8)	0.97 (0.66, 1.43)	0.97 (0.66, 1.44)	82 (39.8)	0.89 (0.60, 1.32)	0.90 (0.60, 1.34)
Myanmar - adults	*n=204*	*n=203*			*n=202*		
On Day 0, n (%)	92 (45.1)	79 (38.9)	0.78 (0.52, 1.15)	0.78 (0.52, 1.15)	66 (32.7)	0.59 (0.40, 0.88)	0.58 (0.38, 0.87)
Between Day 0 - Day 5, n (%)	99 (48.5)	85 (41.9)	0.76 (0.52, 1.13)	0.76 (0.52, 1.13)	76 (37.6)	0.64 (0.43, 0.95)	0.63 (0.42 ,0.94)
Between Day 0 - Day 14, n (%)	102 (50.0)	85 (41.9)	0.72 (0.49, 1.07)	0.72 (0.49, 1.07)	76 (37.6)	0.60 (0.41, 0.90)	0.59 (0.40, 0.89)

Antibiotics prescribed at the facilities included β-lactam molecules (penicillin V, penicillin G, ampicillin, amoxicillin, cloxacillin, dicloxacillin, amoxicillin/clavulanic acid, cefalexin, cefixime and ceftriaxone), macrolides (erythromycin, roxithromycin and azithromycin), quinolones (norfloxacin, levofloxacin, ciprofloxacin), and tetracycline (doxycycline), as well as metronidazole and co-trimoxazole (trimethoprim/sulfamethoxazole). From these, broad-spectrum antibiotics included amoxicillin/clavulanic acid, cefixime, ceftriaxone, azithromycin, levofloxacin and ciprofloxacin.

The commonest antibiotic prescribed was amoxicillin with 72.7% (213/293) and 78.2% (401/513) of the prescriptions at Day 0 in the controls and the combined CRP groups, respectively. Other antibiotics were used in less than 10% of the cases, with cephalosporin and tetracycline/macrolide being the second and third most frequently prescribed antibiotics (8.5% (25/293) in the controls, 7.0% (36/513) in the CRP combined groups, and (7.9% (23/293) in the controls, 5.3% (27/513) in the CRP combined groups respectively). The least prescribed molecules were cotrimoxazole and metronidazole, in less than 3% of the cases (Figure A).

Figure A. Antibiotic prescription in the controls comparing to the CRP combined groups (Group A and Group B) on Day 0

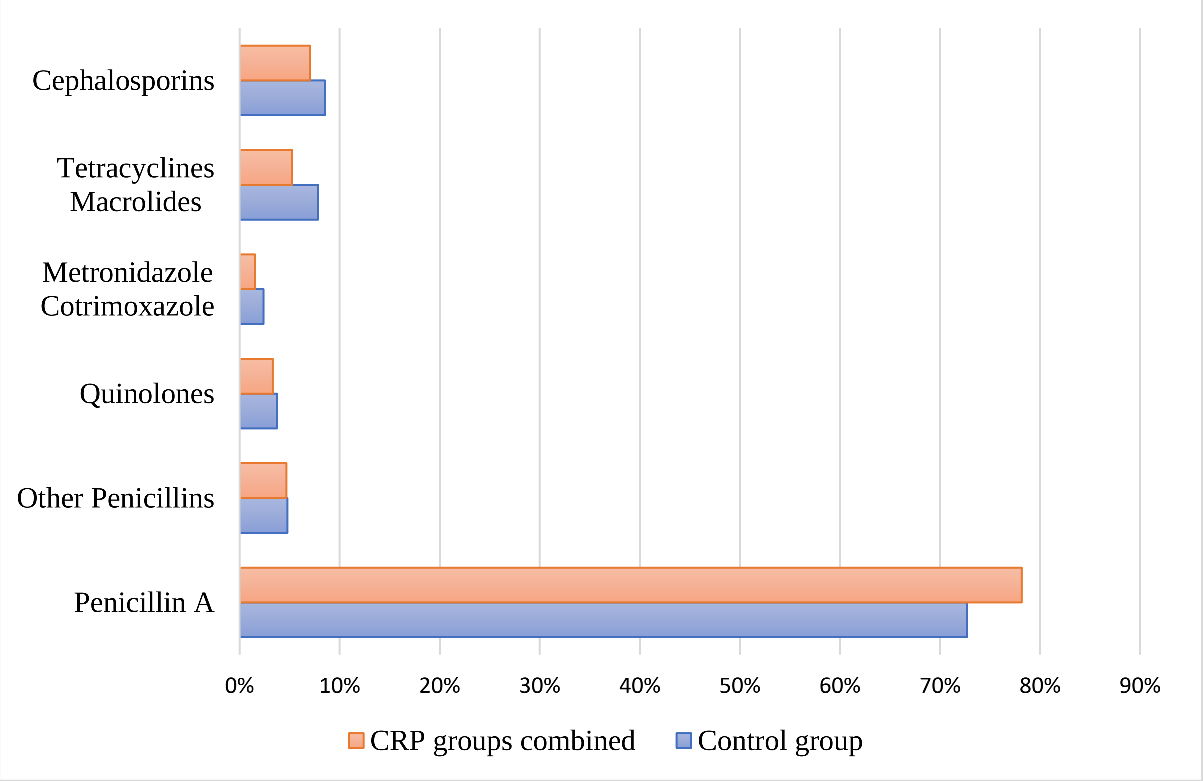

CRP distribution

CRP-levels were below 20 mg/L in around 72% of the patients, while 86% had a CRP below 40 mg/L (figure B). These distributions remained unchanged when considering CRP-levels per country and age category.

Figure B. CRP distribution overall, per country, and per age category

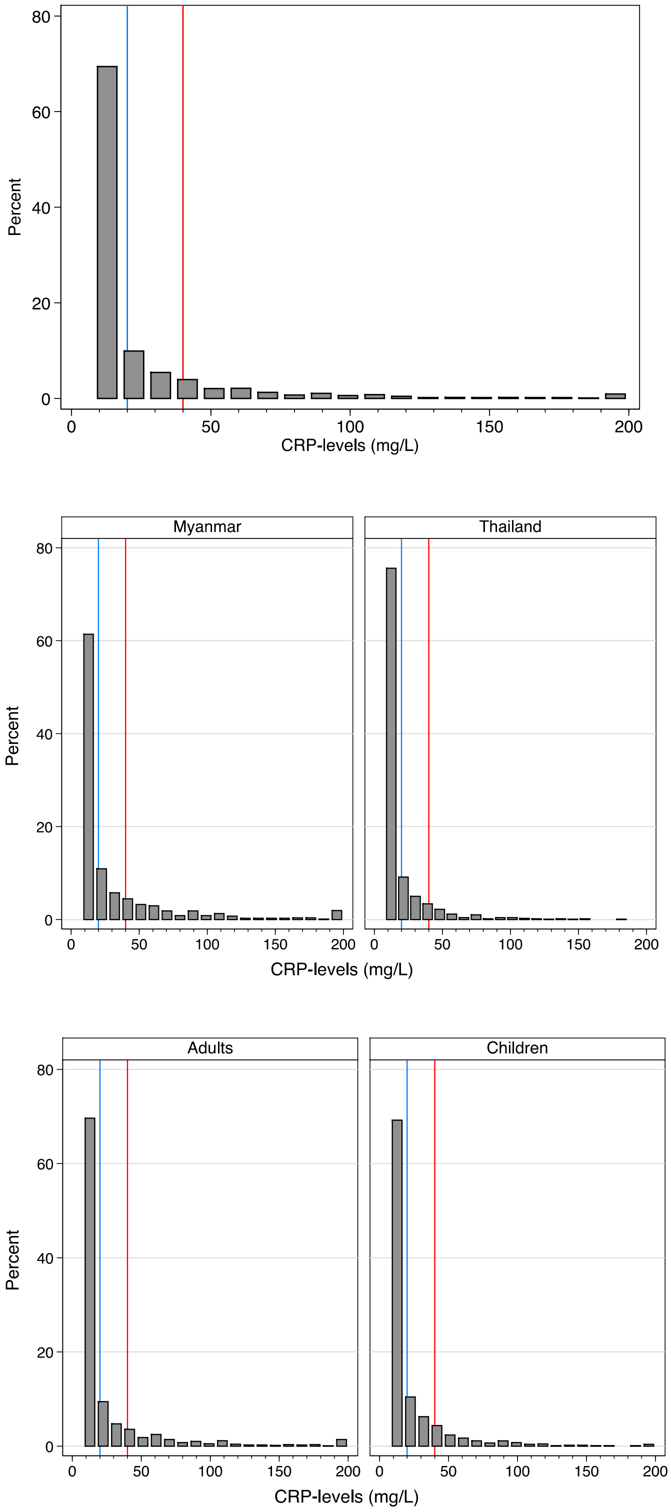

Clinical outcomes

Table 3. Clinical outcomes comparing the controls, Groups A (20mg/L) and B (40mg/L) at Day 5 and Day 14 of the follow- up, overall, per age category and per country

	Controls	Group A	*P-value*	Group B	*P-value*
Overall (Children & Adults - Thailand & Myanmar)					
Persistent symptoms at Day 5, n (%)	276/767 (36.0)	269/764 (35.2)	*0.752*	281/769 (36.5)	*0.821*
Symptom severity at Day 5, median (IQR)	1 (1-1)	1 (1-1)	*0.149*	1 (1-1)	*0.231*
Documented fever at Day 5, n (%)	27/709 (3.8)	22/715 (3.1)	*0.449*	25/726 (3.4)	*0.712*
Elevated CRP at Day 5, n (%)	8/706 (1.1)	8/713 (1.1)	*0.984*	6/726 (0.8)	*0.555*
Persistent symptoms at Day 14, n (%)	34/772 (4.4)	42/760 (5.5)	*0.312*	46/779 (5.9)	*0.181*
Symptom severity at Day 14, median (IQR)	1 (1-1)	1 (1-1)	*0.869*	1 (1-1)	*0.543*
Documented fever at Day 14, n (%)	9/635 (1.4)	11/655 (1.7)	*0.703*	11/661 (1.7)	*0.719*
Unscheduled visits, n (%)	16/807 (2.0)	13/803 (1.6)	*0.583*	22/800 (2.8)	*0.311*
Thailand - children					
Persistent symptoms at Day 5, n (%)	92/192 (47.9)	102/193 (52.9)	*0.333*	98/190 (51.6)	*0.474*
Symptom severity at Day 5, median (IQR)	1 (1-1)	1 (1-1)	*0.226*	1 (1-1)	*0.493*
Documented fever at Day 5, n (%)	2/192 (1.0)	5/188 (2.7)	*0.241*	3/187 (1.6)	*0.631*
Elevated CRP at Day 5, n (%)	1/192 (0.5)	2/188 (1.1)	*0.550*	1/187 (0.5)	*0.985*
Persistent symptoms at Day 14, n (%)	10/195 (5.1)	8/193 (4.2)	*0.645*	13/192 (6.8)	*0.494*
Symptom severity at Day 14, median (IQR)	1 (1-1)	1 (1-1)	*0.371*	1 (1-1)	*0.254*
Documented fever at Day 14, n (%)	1/184 (0.5)	0/185 (0)	*0.315*	0/183 (0)	*0.318*
Unscheduled visits, n (%)	0/195 (0)	0/194 (0)	*1.000*	0/193 (0)	*1.000*
Thailand - adults					
Persistent symptoms at Day 5, n (%)	76/201 (37.8)	82/198 (41.4)	*0.462*	78/196 (39.8)	*0.685*
Symptom severity at Day 5, median (IQR)	1 (1-1)	1 (1-1)	*0.552*	1 (1-1)	*0.252*
Documented fever at Day 5, n (%)	0/200 (0)	0/196 (0)	*1.000*	0/195 (0)	*1.000*
Elevated CRP at Day 5, n (%)	0/200 (0)	0.196 (0)	*1.000*	1/195 (0.5)	*0.311*
Persistent symptoms at Day 14, n (%)	5/201 (2.5)	11/196 (5.6)	*0.113*	8/197 (4.1)	*0.377*
Symptom severity at Day 14, median (IQR)	1 (1-1)	1 (1-1)	*0.324*	1 (1-1)	*0.429*
Documented fever at Day 14, n (%)	0/195 (0)	0/184 (0)	*1.000*	0/188 (0)	*1.000*
Unscheduled visits, n (%)	0/201 (0)	0/200 (0)	*1.000*	1/199 (0.5)	*0.314*
Myanmar - children					
Persistent symptoms at Day 5, n (%)	52/185 (28.1)	41/186 (22.0)	*0.178*	47/195 (24.1)	*0.374*
Symptom severity at Day 5, median (IQR)	1 (1-1)	1 (1-1)	*0.272*	1 (1-1)	*0.320*
Documented fever at Day 5, n (%)	11/170 (6.5)	12/173 (6.9)	*0.863*	14/181 (7.7)	*0.645*
Elevated CRP at Day 5, n (%)	2/169 (1.2)	2/172 (1.2)	*0.986*	1/181 (0.6)	*0.522*
Persistent symptoms at Day 14, n (%)	8/186 (4.3)	8/182 (4.4)	*0.965*	10/196 (5.1)	*0.712*
Symptom severity at Day 14, median (IQR)	1 (1-1)	1 (1-1)	*X*	1 (1-1)	*0.421*
Documented fever at Day 14, n (%)	5/134 (3.7)	6/148 (4.1)	*0.889*	8/153 (5.2)	*0.543*
Unscheduled visits, n (%)	8/207 (3.9)	6/206 (2.9)	*0.593*	15/206 (7.3)	*0.130*
Myanmar - adults					
Persistent symptoms at Day 5, n (%)	56/189 (29.6)	44/187 (23.5)	*0.181*	58/188 (30.9)	*0.796*
Symptom severity at Day 5, median (IQR)	1 (1-1)	1 (1-1)	*0.181*	1 (1-1)	*0.481*
Documented fever at Day 5, n (%)	14/147 (9.5)	5/158 (3.2)	*0.002*	8/163 (4.9)	*0.114*
Elevated CRP at Day 5, n (%)	5/145 (3.5)	4/157 (2.6)	*0.646*	3/163 (1.8)	*0.376*
Persistent symptoms at Day 14, n (%)	11/190 (5.8)	15/189 (7.9)	*0.408*	15/194 (7.7)	*0.449*
Symptom severity at Day 14, median (IQR)	1 (1-1)	1 (1-1)	*0.459*	1 (1-1)	*0.246*
Documented fever at Day 14, n (%)	3/122 (2.5)	5/138 (3.6)	*0.587*	3/137 (2.2)	*0.886*
Unscheduled visits, n (%)	8/204 (3.9)	7/203 (3.5)	*0.800*	6/202 (3.0)	*0.599*

CRP measured at Day 5 was considered high if CRP-levels ≥50mg/L in children, and ≥100mg/L in adults

Symptoms were based on patient's declaration

Documented fever was defined as tympanic temperature>37.5˚C

Results from the per-protocol analysis

As shown in Table 4, there are no differences in the demographic and clinical characteristics between patients included in the per-protocol analysis and those in the intention to treat analysis

Table 4. Day 0 characteristics comparing per-protocol and intention-to-treat populations

Day 0 characteristics	PP population (n=1,191)	ITT population (n=2,410)	*p-value*
**Demographic characteristics**			
Male, n (%)	547 (45.9)	1,106 (45.9)	*0.992*
Age, median (IQR)	11 (4-35)	11 (4-34)	*0.431*
Percentage of children<5-year-old, n (%)	327 (27.6)	650 (27.1)	*0.763*
Presence of comorbidity, n (%)	170 (14.3)	351 (14.5)	*0.856*
Symptom onset (in days), median (IQR)	2 (2-3)	2 (2-3)	*0.821*
≥ 30min to reach the facility, n (%)	265 (22.3)	514 (21.3)	*0.531*
Self-reported antibiotic intake, n (%)	57 (4.8)	129 (5.4)	*0.470*
**Clinical characteristics and self-reported symptoms**			
Documented fever (>37.5˚C), n (%)	512 (43.0)	1,072 (44.5)	*0.351*
Neurological symptoms, n (%)	284 (23.9)	600 (24.9)	*0.491*
Respiratory symptoms, n (%)	950 (79.8)	1,905 (79.1)	*0.616*
Gastrointestinal symptoms, n (%)	271 (22.8)	583 (24.2)	*0.340*
Other symptoms, n (%)	106 (9.1)	211 (9.0	*0.892*
Symptom severity, median (IQR)	1 (1-2)	1 (1-2)	*0.395*

Comorbidities included HIV, chronic hepatitis B or C, cirrhosis, diabetes mellitus, asthma, chronic anaemia, chronic obstructive pulmonary disease, chronic gastritis, congenital heart or kidney disease, chronic alcoholism, dyslipidaemia, G6PD deficiency, hypertension, rheumatic heart disease, thalassaemia, thyroid disease.
Neurological symptoms include headache, confusion, dizziness or hearing loss.
Respiratory symptoms include sore throat, dyspnoea, chest pain, runny nose, or cough.
Gastrointestinal symptoms include nausea, vomiting, diarrhoea, or abdominal pain.
Other symptoms declared were defined by the presence of fever alone or symptoms other than those present in neurological, respiratory, nor gastrointestinal symptoms. Common symptoms in this group included myalgia, arthralgia, jaundice, tiredness, chills, sweating, weight loss, skin eruption, dysuria, or eye redness.

Antibiotic prescribing

Table 5. Antibiotic prescription in the controls, Group A (20mg/L) and Group B (40mg/L); the primary trial outcome (i.e. the prescription of antibiotics from Day 0 to Day 5) is in bold.

	**Controls (n=767)**	**Group A (n=598)**	**RD (95%CI)**	**aOR* (95% CI)**	**Group B (n=593)**	**RD (95%CI)**	**aOR* (95% CI)**
Overall (Children & Adults - Thailand & Myanmar)							
On Day 0, n (%)	283 (36.9)	142 (23.8)	-13.2 (-18.0, -8.3)	0.53 (0.41, 0.67)	86 (14.5)	-22.4 (-26.8, -18.0)	0.28 (0.21, 0.37)
**Between Day 0 - Day 5, n (%)**	**301 (39.2)**	**161 (26.9)**	**-12.3 (-17.3, -7.4)**	**0.56 (0.44, 0.71)**	**112 (18.9)**	**-20.4 (-25.0, -15.7)**	**0.35 (0.27, 0.45)**
Between Day 0 - Day 14, n (%)	306 (39.9)	163 (27.3)	-12.6 (-17.6, -7.7)	0.55 (0.44, 0.70)	116 (19.6)	-20.3 (-25.1, -15.6)	0.35 (0.27, 0.45)
Thailand – children	*n=192*	*n=162*			*n=153*		
On Day 0, n (%)	62 (32.3)	30 (18.5)	-13.8 (-22.7, -4.9)	0.48 (0.29, 0.79)	12 (7.8)	-24.5 (-32.3, -16.6)	0.18 (0.09, 0.35)
**Between Day 0 - Day 5, n (%)**	**66 (34.4)**	**35 (21.6)**	**-12.8 (-22.0, -3.5)**	**0.52 (0.32, 0.85)**	**15 (9.8)**	**-24.6 (-32.8, -16.4)**	**0.21 (0.11, 0.38)**
Between Day 0 - Day 14, n (%)	67 (34.9)	35 (21.6)	-13.3 (-22.5, -4.0)	0.51 (0.31, 083)	15 (9.8)	-25.1 (-33.3, -16.9)	0.20 (0.11, 0.37)
Thailand - adults	*n=201*	*n=145*			*n=137*		
On Day 0, n (%)	63 (31.3)	16 (11.0)	-20.3 (-28.5, -12.1)	0.26 (0.14, 0.49)	12 (8.8)	-22.6 (-30.6, -14.6)	0.21 (0.10, 0.40)
**Between Day 0 - Day 5, n (%)**	**64 (31.8)**	**19 (13.1)**	**-18.7 (-27.2, -10.3)**	**0.31 (0.17, 0.56)**	**15 (11.0)**	**-20.9 (-29.2, -12.6)**	**0.25 (0.14, 0.48)**
Between Day 0 - Day 14, n (%)	64 (31.8)	19 (13.1)	-18.7 (-27.2, -10.3)	0.31 (0.17, 0.56)	16 (11.7)	-20.2 (-28.6, -11.8)	0.27 (0.15, 0.51)
Myanmar - children	*n=185*	*n=145*			*n=156*		
On Day 0, n (%)	71 (38.4)	51 (35.2)	-3.2 (-13.7, 7.3)	0.89 (0.57, 1.41)	33 (21.2)	-17.2 (-26.7, -7.7)	0.44 (0.27, 0.72)
**Between Day 0 - Day 5, n (%)**	**77 (41.6)**	**57 (39.3)**	**-2.3 (-13.0, 8.4)**	**0.91 (0.58, 1.42)**	**46 (29.5)**	**-12.1 (-22.2, -2.1)**	**0.60 (0.38, 0.94)**
Between Day 0 - Day 14, n (%)	78 (42.2)	59 (40.7)	-1.5 (-12.2, 9.2)	0.94 (0.61, 1.46)	49 (31.4)	-10.8 (-20.9, -0.6)	0.64 (0.41, 1.00)
Myanmar - adults	*n=189*	*n=146*			*n=147*		
On Day 0, n (%)	87 (46.0)	45 (30.8)	-15.2 (-25.5, -4.9)	0.52 (0.33, 0.82)	29 (19.7)	-26.3 (-35.9, -16.7)	0.28 (0.17, 0.47)
**Between Day 0 - Day 5, n (%)**	**94 (49.7)**	**50 (34.3)**	**-15.5 (-26.0, -5.0)**	**0.52 (0.34, 0.82)**	**36 (24.5)**	**-25.3 (-35.2, -15.3)**	**0.32 (0.20, 0.52)**
Between Day 0 - Day 14, n (%)	97 (51.3)	50 (34.3)	-17.1 (-27.6, -6.6)	0.49 (0.31, 0.77)	36 (24.5)	-26.8 (-36.8, -16.9)	0.30 (0.19, 0.49)

*aOR were adjusted by site as a random effect

Clinical outcomes

From the 2,410 children and adults recruited, 1,958 (81.2%) complied with both Day 5 and Day 14 follow-up visits, and healthcare providers strictly followed the CRP guidance for prescribing an antibiotic. Concerning clinical outcomes, there was no difference in recovery rates at Day 5 or at Day 14 days of follow-up as showed by Figure C. Log-rank test (p-value 0.502) and HR (0.97, 95% CI 0.87-1.08) did not identify any significant differences between the controls and Group A, as well as with Group B (log-rank test p-value 0.713 and HR 0.99, 95% CI 0.94-1.05).

Figure C: Kaplan-Meier curves of symptoms duration in the controls *versus* Groups A (20mg/L) and B (40mg/L) in per-protocol analysis

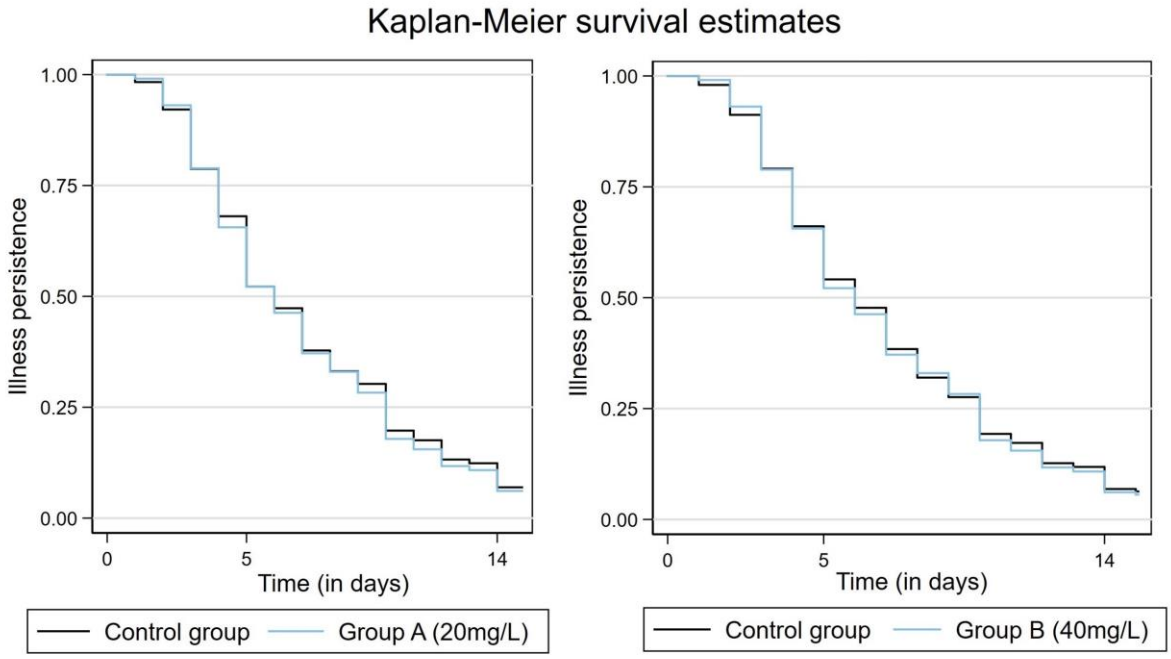

Table 6. Clinical outcomes comparing the controls, Groups A (20mg/L) and B (40mg/L) at Day 5 and Day 14 of the follow- up, overall, per age category and per country

	Controls	Group A	*P-value*	Group B	*P-value*
Overall (Children & Adults - Thailand & Myanmar)					
Persistent symptoms at Day 5, n (%)	272/767 (35.5)	214/598 (35.8)	*0.936*	219/593 (36.9)	*0.622*
Symptom severity at Day 5, median (IQR)	1 (1-1)	1 (1-1)	*0.244*	1 (1-1)	*0.859*
Documented fever at Day 5, n (%)	26/767 (3.4)	17/598 (2.8)	*0.547*	18/593 (3.0)	*0.656*
Elevated CRP at Day 5, n (%)	8/706 (1.1)	8/713 (1.1)	*0.984*	6/726 (0.8)	*0.555*
Persistent symptoms at Day 14, n (%)	34/767 (4.4)	35/598 (5.9)	*0.236*	32/593 (5.4)	*0.410*
Symptom severity at Day 14, median (IQR)	1 (1-1)	1 (1-1)	*1.000*	1 (1-1)	*0.882*
Documented fever at Day 14, n (%)	9 (1.2)	8/598 (1.3)	*0.863*	7/593 (1.2)	*0.964*
Unscheduled visits, n (%)	13/767 (1.7)	10/593 (1.7)	*0.974*	17/593 (2.9)	*0.145*
Thailand - children					
Persistent symptoms at Day 5, n (%)	92/192 (47.9)	91/162 (56.2)	*0.121*	84/153 (54.9)	*0.197*
Symptom severity at Day 5, median (IQR)	1 (1-1)	1 (1-1)	*0.226*	1 (1-1)	*0.493*
Documented fever at Day 5, n (%)	2/192 (1.0)	4/162 (2.5)	*0.285*	2/153 (1.3)	*0.803*
Elevated CRP at Day 5, n (%)	1/192 (0.5)	2/188 (1.1)	*0.550*	1/187 (0.5)	*0.985*
Persistent symptoms at Day 14, n (%)	10/192 (5.2)	8/162 (4.9)	*0.908*	13/153 (8.5)	*0.224*
Symptom severity at Day 14, median (IQR)	1 (1-1)	1 (1-1)	*0.371*	1 (1-1)	*0.254*
Documented fever at Day 14, n (%)	1/192 (0.5)	0/162 (0)	*0.352*	0/153 (0)	*0.371*
Unscheduled visits, n (%)	0/192 (0)	0/162 (0)	*1.000*	0/137 (0)	*1.000*
Thailand - adults					
Persistent symptoms at Day 5, n (%)	76/201 (37.8)	60/145 (41.4)	*0.503*	58/137 (42.3)	*0.404*
Symptom severity at Day 5, median (IQR)	1 (1-1)	1 (1-1)	*0.552*	1 (1-1)	*0.252*
Documented fever at Day 5, n (%)	0/201 (0)	0/145 (0)	*1.000*	0/137 (0)	*1.000*
Elevated CRP at Day 5, n (%)	0/200 (0)	0.196 (0)	*1.000*	1/195 (0.5)	*0.311*
Persistent symptoms at Day 14, n (%)	5/201*(2.5)*	8/145 (5.5)	*0.144*	3/137 (2.2)	*0.860*
Symptom severity at Day 14, median (IQR)	1 (1-1)	1 (1-1)	*0.324*	1 (1-1)	*0.429*
Documented fever at Day 14, n (%)	0/201 (0)	0/145 (0)	*1.000*	0/137 (0)	*1.000*
Unscheduled visits, n (%)	0/201 (0)	0/145 (0)	*1.000*	1/137 (0.5)	*0.314*
Myanmar - children					
Persistent symptoms at Day 5, n (%)	50/185 (27.0)	30/145 (20.7)	*0.167*	32/156 (20.5)	*0.137*
Symptom severity at Day 5, median (IQR)	1 (1-1)	1 (1-1)	*0.272*	1 (1-1)	*0.320*
Documented fever at Day 5, n (%)	11/185 (6.0)	9/145 (6.2)	*0.940*	11/156 (7.1)	*0.707*
Elevated CRP at Day 5, n (%)	2/169 (1.2)	2/172 (1.2)	*0.986*	1/181 (0.6)	*0.522*
Persistent symptoms at Day 14, n (%)	8/185 (4.3)	7/145 (4.8)	*0.825*	7/156 (4.5)	*0.930*
Symptom severity at Day 14, median (IQR)	1 (1-1)	1 (1-1)	*X*	1 (1-1)	*0.421*
Documented fever at Day 14, n (%)	5/185 (2.7)	5/145 (3.5)	*0.858*	6/156 (3.9)	*0.629*
Unscheduled visits, n (%)	5/185 (2.7)	3/145 (2.1)	*0.710*	14/156 (9.0)	*0.012*
Myanmar - adults					
Persistent symptoms at Day 5, n (%)	54/189 (28.6)	33/146 (22.6)	*0.209*	45/147 (30.6)	*0.700*
Symptom severity at Day 5, median (IQR)	1 (1-1)	1 (1-1)	*0.181*	1 (1-1)	*0.481*
Documented fever at Day 5, n (%)	13/189 (6.9)	4/146 (2.7)	*0.065*	5/147 (3.4)	*0.096*
Elevated CRP at Day 5, n (%)	5/145 (3.5)	4/157 (2.6)	*0.646*	3/163 (1.8)	*0.376*
Persistent symptoms at Day 14, n (%)	11/189 (5.8)	12/146 (8.2)	*0.397*	9/147 (6.1)	*0.917*
Symptom severity at Day 14, median (IQR)	1 (1-1)	1 (1-1)	*0.459*	1 (1-1)	*0.246*
Documented fever at Day 14, n (%)	3/189 (1.6)	3/146 (2.1)	*0.832*	1/147 (0.7)	*0.375*
Unscheduled visits, n (%)	8/189 (4.2)	7/146 (4.8)	*0.805*	3/147 (2.0)	*0.263*
